# Nociceptor mechanisms underlying pain and bone remodeling via orthodontic forces: toward no pain, big gain

**DOI:** 10.3389/fpain.2024.1365194

**Published:** 2024-02-22

**Authors:** Sheng Wang, Ching-Chang Ko, Man-Kyo Chung

**Affiliations:** ^1^ Division of Orthodontics, College of Dentistry, The Ohio State University, Columbus, OH, United States; ^2^ Department of Neural and Pain Sciences, School of Dentistry, University of Maryland Baltimore, Baltimore, MD, United States; ^3^ Center to Advance Chronic Pain Research, University of Maryland Baltimore, Baltimore, MD, United States

**Keywords:** orthodontic pain, nociceptors, transient receptor potential (TRP) channel, alveolar bone remodeling, neuropeptides

## Abstract

Orthodontic forces are strongly associated with pain, the primary complaint among patients wearing orthodontic braces. Compared to other side effects of orthodontic treatment, orthodontic pain is often overlooked, with limited clinical management. Orthodontic forces lead to inflammatory responses in the periodontium, which triggers bone remodeling and eventually induces tooth movement. Mechanical forces and subsequent inflammation in the periodontium activate and sensitize periodontal nociceptors and produce orthodontic pain. Nociceptive afferents expressing transient receptor potential vanilloid subtype 1 (TRPV1) play central roles in transducing nociceptive signals, leading to transcriptional changes in the trigeminal ganglia. Nociceptive molecules, such as TRPV1, transient receptor potential ankyrin subtype 1, acid-sensing ion channel 3, and the P2X3 receptor, are believed to mediate orthodontic pain. Neuropeptides such as calcitonin gene-related peptides and substance P can also regulate orthodontic pain. While periodontal nociceptors transmit nociceptive signals to the brain, they are also known to modulate alveolar bone remodeling in periodontitis. Therefore, periodontal nociceptors and nociceptive molecules may contribute to the modulation of orthodontic tooth movement, which currently remains undetermined. Future studies are needed to better understand the fundamental mechanisms underlying neuroskeletal interactions in orthodontics to improve orthodontic treatment by developing novel methods to reduce pain and accelerate orthodontic tooth movement—thereby achieving “big gains with no pain” in clinical orthodontics.

## 1 Introduction

The primary objective of orthodontic treatment is to correct malocclusion by moving teeth within the alveolar bone. Orthodontic tooth movement due to the application of force is often accompanied by side effects such as root resorption, periodontal disease, pulp reaction, and orthodontic pain. Among these, pain is the leading complaint, with 90% of patients affected. Orthodontic pain is also one of the most common reasons for reduced patient compliance and treatment discontinuation (2–4), with approximately 30% of patients having considered stopping treatment due to pain (5). Moreover, orthodontic pain decreases patient health-related quality of life by impairing daily-life activities such as eating and talking (6, 7). Unfortunately, the clinical management of orthodontic pain is unsatisfactory. Orthodontists often recommend treatment with nonsteroidal anti-inflammatory drugs (NSAIDs) and most of the randomized controlled trials studies orthodontic pain compared other interventions with NSAIDs (8), which adversely affect the efficiency of tooth movement (9). Therefore, it is essential to improve our understanding of the mechanisms underlying orthodontic pain at the molecular and cell biology levels to develop new therapies for orthodontic pain that do not interfere with tooth movement.

**Figure 1:**
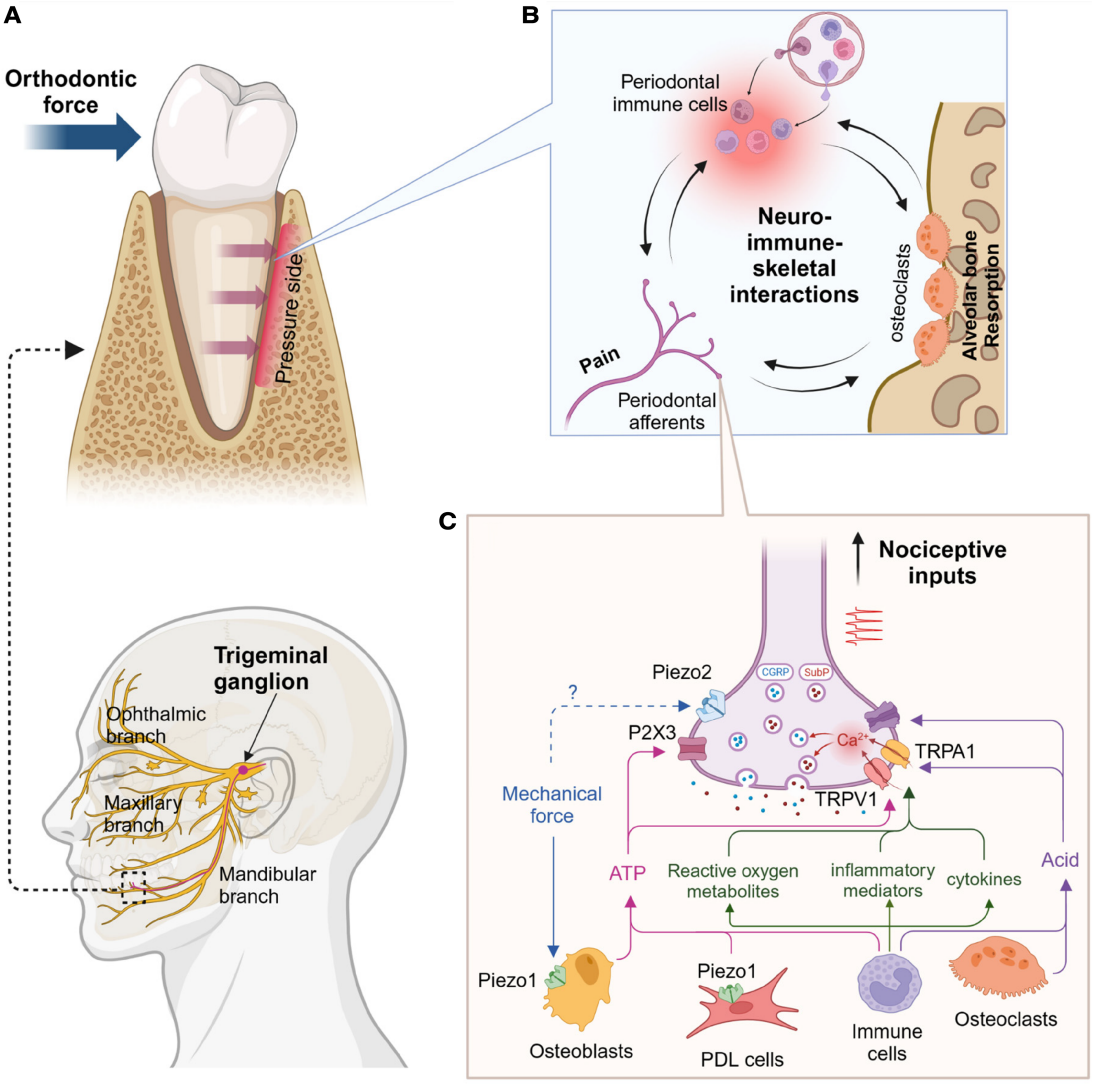
Proposed primary afferent mechanisms of orthodontic pain. (**A**) Anatomy of trigeminal afferents (bottom) and mandibular premolar under orthodontic pressure (top). Alveolar bone in the pressure side undergoes resorption. (**B**) Hypothetical neural-immune-skeletal interactions at the site of orthodontic pressure. Primary afferent terminals and immune cells within periodontal ligaments can regulate alveolar bone through the modulation of osteoclasts, which can also affect both neural and immune system. (**C**) Proinflammatory cytokines (e.g., interleukin 1β, tumor necrosis factor), inflammatory mediators (e.g., prostaglandin E2, bradykinin), reactive oxygen metabolites (e.g., hydrogen peroxide, 8-hydroxy-2'-deoxyguanosine) can activate and sensitize transient receptor potential vanilloid subtype 1 (TRPV1) and transient receptor potential ankyrin subtype 1 (TRPA1). Their activation induces calcium influx into nerve terminals and triggers exocytosis of the vesicles containing calcitonin gene-related peptide (CGRP) and substance P (SubP). Mechanical forces can activate Piezo1 in periodontal ligament (PDL) cells and osteoblasts, which induces ATP release likely activating P2X3 receptor. Immune cells or osteoclasts can increase acids to activate acid-sensing ion channels 3 (ASIC3). Concerted activation of these cationic ion channels in the terminal can generate action potential, which is transmitted into brain and leads to nociception. Created using Biorender.

Orthodontic pain is primarily due to acute inflammation. The self-limiting pain usually occurs approximately four hours after placing the initial archwire, peaks around day one, gradually decreases after three to seven days, and returns to the baseline level in a month (1, 10, 11). During the first week, the patient's quality of life is affected in terms of difficult eating, impaired talking, and oral ulcers. Once orthodontic forces are applied to the teeth, compression and tension zones occur in the periodontal ligament around the affected teeth. On the compression side, inflammatory mediators are released from resident immune cells, which further recruit circulating immune cells (12–18). This aseptic inflammation is an essential element for inducing osteoclastogenesis, leading to orthodontic tooth movement on the compression side (19). Primary afferent terminals within the periodontal ligament [a subpopulation of trigeminal ganglia (TG) neurons] and pain pathways in the brain have been determined. Nociceptors at the periodontal ligament transduce mechanical stimulation from orthodontic forces and chemical stimuli via inflammation, and nociceptive signals during orthodontic treatment are transmitted through the TG (Figure 1A). In this review, we highlight recent progress in understanding primary afferent mechanisms leading to orthodontic pain. As psychological factors such as anxiety, stress, and environmental factors can also affect orthodontic pain perception (20, 21), understanding central pathways involved in orthodontic pain is also crucial–but has been reviewed elsewhere (22). Orthodontic forces induce bone remodeling, which involves the contributions of immune cells and their regulations of bone cells in periodontium. This process is modulated by sensory nerves and, therefore, nociceptive nerves at the site of orthodontic force application should contribute to orthodontic bone remodeling as well as orthodontic pain (Figures 1A,B).

## 2 Clinical factors influencing orthodontic pain and its management

Various patient-related factors, including the patient's age (1, 23, 24), gender (1, 2, 10, 23–27), race (23, 28), and baseline pain threshold (1, 23), affect orthodontic pain perception. However, another systematic review found no effects of age and sex on orthodontic pain (29).

The types of orthodontic appliances, such as separator placements, archwire placements, fixed and removable appliances, and growth modification appliances (including expanders and headgear) also cause varying levels of pain and discomfort (4). Currently, with the increasing use of self-ligation brackets and clear aligners, clinicians have found that self-ligation brackets produce less pain during extraction space closure compared to conventional brackets (30). Clear aligners produces lower level of pain and anxiety than fixed appliances during the first few days of treatment (and for up to 3 months) (31).

Pain and discomfort occur once the initial archwire is placed and last until bracket debonding. At the debonding appointment, the removal force is greater for metal brackets than other types, and pain tends to be greater in the anterior segments than posterior ones in the upper and lower dental arch (32).

Currently, the most effective method to decrease orthodontic pain is via analgesics. NSAIDs are often used to relieve orthodontic pain by blocking the formation of prostaglandins (23, 28). Pharmacological treatments are beneficial because of their rapid and reasonably effective pain relief along with easy over-the-counter access. However, pharmacological treatments may potentially slow down the rate of tooth movement, cause various adverse side effects, and offer only transient pain alleviation. While pharmacological treatment is a mainstay for orthodontic pain management in clinical orthodontics, there is growing interest in exploring non-pharmacological interventions, such as low-level laser therapy (LLLT), vibratory devices, chewing adjuncts, brainwave music, cognitive behavioral therapy, and post-treatment communication in the form of text messages (33–35). The non-pharmacological approaches can be advantageous due to their low risk of adverse side effects. However, the analgesic efficacy of these non-pharmacological approaches is inconclusive due to low evidence quality—although LLLT was shown to reduce pain up to seven days after initial archwire placement in a study (34, 36). A transcutaneous electrical nerve stimulation-based device also shows analgesic efficacy, which need more validation (37).

## 3 Primary afferent contributions to orthodontic pain

### 3.1 Peripheral nerve innervations in the periodontium

The periodontium is well-innervated by primary afferent neurons. Early ultrastructural studies identified myelinated and unmyelinated mechanosensitive nerve terminals within the periodontal ligaments of humans and experimental animals (38, 39). The periodontium is projected by multiple mechanosensory afferents whose cell bodies are located in the TG and mesencephalic trigeminal nucleus (40). Morphologically, several kinds of nerve endings have been identified in the periodontal ligaments of rat molars: nerve fibers with large Ruffini-like endings, bundles of free nerve endings of unmyelinated axons, and free myelinated axons (41). Single fiber recordings have shown nerve terminals innervating the periodontal ligament that include rapidly and slowly adapting Aβ-fibers, medium-diameter Aδ-fibers, and small-diameter C-fibers (42–45). Retrograde labeling of periodontal afferents has shown that periodontal trigeminal afferents comprise small- to medium-diameter neurons. Neurochemically, approximately 25% of periodontal afferents contain calcitonin gene-related peptide (CGRP) and transient receptor potential vanilloid subtype 1 (TRPV1). Periodontal afferents express various chemical receptor molecules, which transmit noxious chemical stimuli to the brain as a pain sensation. They also express mechanosensitive ion channels, which transduce sensations of pressure and stretching to the brain. These receptors and molecules, including TRPV1 (46–51), transient receptor potential ankyrin 1 (TRPA1) (47, 48, 52), Piezo1 (48, 53–57), Piezo2 (48, 53), acid-sensing ion channel 3 (ASIC3) (58–61), purinergic receptor (e.g., P2X3) (62, 63), are discussed below.

Sympathetic nerves, the component of the autonomic nervous system, originate from the superior cervical ganglion, are closely associated with the vasculature within the periodontium and regulate blood flow. Sympathetic nervous system contributes to wound healing in periodontal tissues (64, 65). While sympathetic nervous system regulates orthodontic tooth movement (66), evidence supporting the roles of sympathetic nerves in orthodontic pain is lacking and this review will focus on nociceptive primary afferents.

### 3.2 Periodontal nociceptors

Orthodontic pain is initiated by orthodontic force applied to the teeth. This force mechanically irritates periodontal tissues, which subsequently induces a cascade of vascular and chemical reactions, possibly affecting pain and bone remodeling (reviewed in Long, et al. and Tang, et al. (7, 67). The International Association for the Study of Pain (IASP) defines nociceptor as “a high-threshold sensory receptor of the peripheral somatosensory nervous system that is capable of transducing and encoding noxious stimuli.” The term refers to the nerve endings that initiate the transduction of noxious stimuli. Periodontal nociceptors are nerve endings that transduce noxious stimuli in the periodontium. Single-fiber recordings in rats have shown that periodontal nociceptors in the lower incisors are composed of thinly myelinated Aδ or unmyelinated c-fibers. Unlike nociceptors in the oral mucosa or tooth pulp (68), periodontal nociceptors are mostly Aδ (approximately 90%) rather than c nociceptors (69). In rat molars, most periodontal ligament units rapidly adapt, and two-thirds are Aδ fibers (44). The Aδ nociceptors may mediate immediate, sharp pain upon the application of orthodontic forces. This initial pain is followed by delayed pain, a major component of patient discomfort. Pain intensity gradually increases four hours following the application of orthodontic forces, peaking after 24 h, and lasts for days (1, 10, 23, 27, 70). Orthodontic force loading in rat molars reduces the mechanical threshold in behavioral assays and action potential conduction velocity in single-fiber recordings after 3–14 days (71). Such delayed pain may be derived from peripheral sensitization of the periodontal nociceptor terminals, as orthodontic forces induce inflammation in the periodontium to produce an array of inflammatory mediators and cytokines (7).

### 3.3 TRPV1-expressing nociceptors in orthodontic pain

Retrograde labeling by injecting a tracer into mouse gingiva has demonstrated that periodontal afferents are primarily small- and medium-diameter neurons (46, 72). Among these, 23% are CGRP-positive and 28% are TRPV1-positive. Non-peptidergic nociceptors binding to isolectin B4 are rare among periodontal afferents. Although TRPV1-expressing afferents mediate heat pain in the skin, they also mediate mechanical hyperalgesia from deep tissues such as muscles and joints (73–76). TRPV1-expressing afferents also play a critical role in orthodontic pain. Chemical ablation of TRPV1-expressing nociceptors from the TG of mice by injecting resiniferatoxin (RTX) into the TG substantially reduces orthodontic pain-like behaviors. Orthodontic forces increase mouse grimace scale scores and reduce bite force after one and three days, which is partially prevented by the ablation of TRPV1-expressing TG neurons (46).

TRPV1-expressing nociceptors can also contribute to the development of orthodontic pain. As TRPV1 and neuropeptides are highly colocalized in periodontal afferents (46, 72), and nerve terminals containing neuropeptides such as CGRP and substance P (SP) are projected into periodontal ligaments (77, 78), the activation of TRPV1-expressing nerve terminals by orthodontic forces can induce the release of neuropeptides such as CGRP or SP into the periodontal tissues. These neuropeptides play crucial roles in vasodilation and the recruitment of immune cells to damaged tissue (79). This neurogenic inflammation should then initiate sterile inflammation, leading to orthodontic tooth movement and peripheral sensitization. Therefore, interactions of periodontal nociceptors, immune cells, and bone cells should be critical for orthodontic tooth movement and pain (Figure 1B). The roles of neuropeptides in orthodontic pain are discussed in the sections below.

The results of a recent study indicate that TRPV1-expressing afferents contribute to the plastic changes in gene expression within the TG after applying an orthodontic force in mice (50). Orthodontic forces changed the expression of >1,200 genes in the TG after two days. These genes include those implicated in pain processing, such as neuropeptides (*Adcyap1* and *Gal*), neurotrophins (*Bdnf*), neurotrophin receptors (*Gfra1*), cytokines (*Csf1* and *Cx3cl1*), cytokine receptors (*Cxcr4* and *Tnfrsf1a*), transcription factors (*Atf3* and *Sox11*), and ion channels (*Trpa1* and *Trpv2*). The contribution of these gene changes to orthodontic pain needs to be determined in future studies. Gene ontology analyses have shown increased cholesterol biosynthesis processes and decreased organization of connective tissue and extracellular matrix (50). Genes associated with synaptic organization and overall ion channel activities, especially voltage-gated sodium and potassium channels, are downregulated. The biological pathways and differentially expressed genes in the TG after orthodontic tooth movement resemble early changes following peripheral nerve injury rather than craniofacial inflammation. The implication of this finding is unclear, but orthodontic forces may induce injury to the afferent terminals in the periodontal ligament, which leads to transcriptomic changes in the TG similar to those following nerve injury. Interestingly, in mice with RTX injections into the TG to ablate TRPV1-expressing sensory neurons, the transcriptomic changes in the TG by orthodontic forces are eliminated (50). However, this does not imply that orthodontic force-induced transcriptomic changes are exclusively confined to TRPV1-expressing nociceptors. Instead, neuronal inputs through TRPV1-expressing nerves drive TG-wide transcriptomic changes after an orthodontic force. Therefore, TRPV1-expressing neurons are critical for transducing nociceptive inputs and inducing neural plasticity in the TG, which should contribute to orthodontic pain development.

## 4 Role of ion channels in periodontal nociceptors in orthodontic pain

### 4.1 Transient receptor potential ion channels

TRPV1 is enriched in peptidergic afferents, most of which are polymodal afferents (73). Its activation mediates the influx of cations into the nerve terminals, followed by the firing of action potentials, which leads to burning pain. Activation of TRPV1 also mediates the Ca^2+^ influx, which produces the release of neuropeptides from afferent terminals. Inflammatory mediators enhance TRPV1 function. Activation of receptors of multiple inflammatory mediators such as prostaglandins, bradykinin, and ATP invokes the activation of protein kinases. This, in turn, phosphorylates TRPV1 to enhance the function of TRPV1, which could increase pathological pain (73, 80, 81). Therefore, local inflammation in the periodontium following orthodontic tooth movement likely enhances the activation of TRPV1, which increases the release of neuropeptides. TRPV1 expressed in afferent terminals within the periodontal ligaments may also be phosphorylated by hypoxia-inducible factor-1*α*, activated by local hypoxia within the periodontal ligament (82, 83). Tissue inflammation has upregulated the expression of TRPV1 in nociceptive afferents in multiple preclinical models. Indeed, TRPV1 is upregulated in the TG following experimental tooth movement in rats, where the upregulation of TRPV1 peaked after one day and returned close to baseline after a week, which correlated with tooth movement-induced nocifensive behaviors such as face-grooming (84). Local administration of TRPV1 antagonist in the periodontium reduces tooth movement-induced nocifensive behaviors (85). Genetic knockout of TRPV1 attenuates spontaneous and bite-evoked pain after applying orthodontic forces (46). Knockdown of TRPV1 in the TG also reduces pain-like behaviors upon the application of orthodontic forces (86). Thus, TRPV1 in periodontal nociceptors mediates burning pain from orthodontic forces, and the inhibition of TRPV1 may attenuate pain related to orthodontic tooth movement.

TRPA1, which is activated by mustard oil and endogenous electrophiles such as hydrogen peroxide (H_2_O_2_) (73, 87, 88), also contributes to neuropeptide release and neurogenic inflammation. Thirty percent of TRPV1-expressing neurons express TRPA1, while up to 97% of TRPA1-expressing sensory neurons express TRPV1 (89), and the calcium influx TRPV1 causes can lead to the activation of TRPA1 (90). TRPA1 and TRPV1 play an integral role in neurogenic inflammation and pain after noxious stimuli due to their high co-expression, and the calcium influx caused by one can lead to the activation of the other (91–93). TRPA1 contributes to mechanical paresthesia caused by trigeminal neuropathic pain in mice downstream of oxidative stress (94). Oxidative stress may induce pain via the activation/sensitization of TRPA1 in the periodontium in a rat tooth-movement model (52). The tooth movement induced nociceptive behaviors, such as facial wiping, which matched the level of 8-hydroxy-2'-deoxyguanosine, an oxidative stress marker in the periodontal ligament and dental pulp. This change gradually diminished to the original level, which was related to decreases in oxidative stress, probably due to the remodeling of the periodontal ligament and alveolar bone (52). Orthodontic force-induced local hypoxia and reduced fluid flow occur in both periodontal tissue and dental pulp (95). Ischemia increases the intracellular Ca^2+^ concentration by increasing the concentration of protons and promoting TRPA1 activation (96, 97). Ca^2+^ influx through TRPA1 in sensory neuronal soma and nerve terminals can induce the release of neuropeptides, promoting the inflammatory periodontal tissue response. The expression of TRPV1 increases in the TG within a day (as an early response), while the upregulation of TRPA1 gradually increases from day one to day three as a late response during experimental tooth movement in rats (47). Therefore, the combined inhibition of TRPV1 and TRPA1 can additively attenuate orthodontic pain.

### 4.2 Mechanosensitive ion channels

Piezo1 and Piezo2 have emerged as important mediators in various aspects of mechanotransduction (98). They convert applied force into electrochemical signals critical for proprioception, touch, and mechanical pain. Piezo1 is expressed in non-neuronal tissues, such as the vasculature, bone, and heart, and has been shown to sense different mechanical stresses, such as compression and stretch. Piezo2 is exclusively expressed in sensory neurons and is related to touch sensation and mechanical pain.

Research concerning Piezo1 and Piezo2 in orthodontic tooth movements has rapidly developed from *in vitro* to *in vivo* studies. After exposure to mechanical loading in primary human periodontal ligament cells, the expression of Piezo1 and markers for osteoclastogenesis, such as receptor activator of nuclear kB ligand and cyclooxygenase-2, were significantly increased (99). Furthermore, grammostola mechanotoxin 4 (GsMTx4), a Piezo1 inhibitor, blocked osteoclastogenesis (99), suggesting Piezo1 contributes to mechanical stress-induced osteoclastogenesis. In murine cementoblastic cells, the expression of Piezo1 was decreased under a static compression force (100). Compression force also decreases cementoblastic genes such as osteoprotegerin, osteopontin, osteocalcin, and protein tyrosine phosphatase-like member A (100), suggesting that Piezo1 may contribute to the remodeling of cementum during tooth movement. When hydrostatic pressure is applied to mesenchymal stem cells (MSCs), Piezo1 is activated and plays a role in the cell fate determination of MSCs (either osteoblast or adipocyte differentiation) by regulating bone morphogenetic protein 2 expression (101). However, activation of Piezo1 by its agonist, Yoda1, induces a Ca^2+^ response and activates cationic currents in osteoblastic cells, followed by reduced proliferation that is reversed by the knockdown of Piezo1 (102). Furthermore, the C-terminus of Piezo1, which contains the R-Ras binding domain, plays an essential role in Ca^2+^ influx and activation of the ERK1/2 signaling pathway, suggesting that this domain is crucial for the mechanotransduction of osteoblastic differentiation in MSCs (103). Piezo1 also plays a role in macrophage infiltrates in the periodontal ligament via the Piezo1-AKT/GSK3b signaling-Cyclin D1 axis during tooth movement (104) and mediating both osteogenesis and osteoclastic activities on the tension side during orthodontic tooth movement (54, 57, 105). However, *in vivo* tooth movement models, the function of Piezo1 is controversial. In rats, orthodontic tooth movement was modestly reduced by the local injection of GsMTx4 into the alveolar bone (57). In contrast, tooth movement was not altered in Piezo1 conditional knockouts in mineralized tissue cells by *Dmp1*-cre (106).

The roles of Piezo1 and Piezo2 in orthodontic pain are undetermined. Piezo1 is functionally expressed in the TG and dorsal root ganglion neurons in rodents, a subset of which co-express TRPV1 (107, 108). Yoda1, a Piezo1 agonist, induces the release of CGRP from TG neurons (107). Therefore, Piezo1 in peptidergic afferents may transduce mechanical pain during orthodontic tooth movement. However, evidence that Piezo1 in sensory neurons mediates mechanical pain is lacking. Instead, Piezo1 expressed in non-neuronal cells may indirectly contribute to pain. For example, Piezo1 expressed in keratinocytes regulates mechanotransduction (109) and, when expressed in odontoblasts, it may mediate nociceptive signaling through the release of ATP by the activation of P2X3 in sensory neurons (110). The activation of Piezo1 in human periodontal ligament cells by Yoda1 increases intracellular Ca^2+^ and extracellular ATP (55). Mechanically stimulated ATP released from human periodontal ligament cells is inhibited by GsMTx4 or the knockdown of Piezo1 (55), suggesting that Piezo1 in periodontal ligament cells may contribute to purinergic signaling during orthodontic tooth movement, leading to pain.

Piezo2 is expressed more abundantly in sensory neurons, including low-threshold mechanosensitive neurons, as well as Aδ and C nociceptors (111, 112). Piezo2 mediates innocuous touch sensation and mechanical hyperalgesia following inflammation and nerve injury (111, 112). Piezo2 is also expressed in TRPV1-expressing nociceptors and mediates corneal mechanical pain, visceral mechanical hypersensitivity, and mechanical hyperalgesia in knee joint osteoarthritis (113–115). Therefore, Piezo2 in TRPV1-expressing TG neurons may mediate orthodontic pain; however, this needs to be determined in future studies.

### 4.3 Other nociceptive ion channels

Acid-sensing ion channels sense extracellular acidification. Among this family, ASIC3 is predominantly expressed in the peripheral nervous system and contributes to nociception. In rats, 26% of ASIC3-expressing TG neurons co-express CGRP (116). Orthodontic force could induce localized tissue acidosis at the site of compression. Tissue inflammation can lead to the accumulation of lactic acids, which causes prolonged acidosis (117). Orthodontic forces induce the occlusion of the blood vessels in the periodontal ligament on the compression side, which leads to hypoxia (118). Hypoxia can then induce tissue acidosis and regulate bone metabolism (119). The suppression of orthodontic pain-like behaviors by a pharmacological inhibitor or genetic knockdown against ASIC3 has been reported in an orthodontic tooth movement model in rats (58, 60). Orthodontic force-induced upregulation of nerve growth factor in the periodontium, which is retrogradely transported to the TG, induces increased expression of ASIC3 (61). In another study, inserting an elastic between the molars of rats acidified gingival crevicular fluid (from pH 7.4 to 7) after one day (59). Buffering periodontal acidification by repeated injections of phosphate-buffered saline or a periodontal injection of the inhibitor of ASIC3 decreased mechanical hyperalgesia in the facial skin after elastic insertion. However, in that study, elastic insertion did not change the expression of ASIC3 in the TG but increased the phosphorylation of ASIC3 in the periodontal tissues.

Extracellular ATP is an important signaling coordinator of cellular responses to mechanical stimulation in bone (120). It activates a group of purinergic receptors, of which P2X3 has been implicated in orthodontic pain. Although the P2X3 receptor is more often expressed in non-peptidergic TG neurons, a subset of P2X3-expressing neurons also co-express CGRP and SP (121). Orthodontic tooth movement upregulates the P2X3 receptor in the TG, while an inhibitor of P2X3 reduces orthodontic pain-like behaviors in rats (62). Orthodontic tooth movement in rats upregulates the nociceptin/orphanin FQ-opioid receptor-like receptor pathway in the TG, which exacerbates pain-like behaviors and likely mediates P2X3 upregulation in TG neurons (122, 123). Interestingly, the exposure of mice to static magnetic fields decreases orthodontic pain-like behaviors, accompanied by a decrease in the expression of P2X3 (124), supporting the roles of P2X3 in orthodontic pain.

## 5 Role of neuropeptides in orthodontic pain

SP and CGRP are the primary neuropeptides studied in orthodontic pain. The increased expression levels in the periodontal ligaments and dental pulp during tooth movement are positively correlated with orthodontic pain, clarifying the role of neurogenic inflammation in early injury response (125). Increased expression levels of CGRP and SP occur during tooth movement and are evident for a considerable time after movement has ended (78). Furthermore, neuropeptides stimulate human pulp fibroblasts to produce large amounts of interleukin-1β (IL-1β), interleukin-6, and tumor necrosis factor-α (TNF-α) during orthodontic tooth movement (126).

CGRP is expressed in the TG, and the central terminals of primary afferents are terminated in the trigeminal subnucleus caudalis (127). Inflammation induced by tooth movement sensitizes TRPV1 and TRPA1 in the primary afferent terminals (52). Activation of TRPV1, as an early response, and TRPA1, as a late response, results in CGRP release at the end of neuronal axons in vesicles through exocytosis (47). CGRP released from periodontal afferent terminals into the periodontal tissue plays the roles of vasodilator and transmitter for nociceptive information. Local injection of a CGRP receptor antagonist into the periodontium reduces pain-like behaviors by rats in experimental tooth movement (128). CGRP also mediates neuron-glia crosstalk by upregulating the expression of nitric oxide in the p38 signaling pathway in glial cells, accelerating the release of signal molecules that stimulate neurons and promote orofacial pain (129).

SP is produced in peptidergic sensory neurons in the TG and is secreted from their axons after Ca^2+^ influx (130). SP plays an essential role in bone remodeling and orthodontic pain during tooth movement. Orthodontic tooth movement activates nociceptors in periodontal tissues and leads to the release of SP from primary afferents. Moreover, tooth movement increases the Ca^2+^ influx after the activation of cation channels, causing SP release. SP promotes local inflammation by increasing the permeability of blood vessels (131, 132). It also promotes the secretion of inflammatory cytokines, including IL-1β and TNF-α through the neurokinin-1 receptor, enhances the phosphorylation of the mitogen-activated protein kinase signaling pathway to activate crosstalk between neurons and glial cells, and speeds the inflammatory process on the compression side of tooth movement (94, 133–135). Inflammation further promotes bone remodeling and tooth movement. Exogenous SP can promote alveolar bone remodeling and accelerate orthodontic tooth movement (136). However, it also increases inflammatory mediators released from immune cells, stimulating TRPV1 nociceptors conducting pain signals to the brain (137, 138). Ibuprofen decreases SP levels in gingival fluid and scores of visual analog scale one day following initial archwire placement (139).

Proposed primary afferent mechanisms of orthodontic pain is summarized in Figure 1C.

## 6 Bone remodeling and the interactions between bone and nociceptors

### 6.1 Bone remodeling in tooth movement

Bone remodeling is an important biological metabolic function that maintains healthy bone density and homeostasis and serves as a mechanism to regulate orthodontic tooth movement. Among the skeletal system, alveolar bone is under the most active bone remodeling (140–143). According to the pressure-tension theory, alveolar-bone-coupled remodeling events are orchestrated by the activities of osteoclasts, osteoblasts, and osteocytes in the periodontal ligament space and alveolar bone after a force is applied to teeth by archwires.

Osteoclasts are specialized cells originating from hematopoietic progenitors, which can resorb host bone tissue (144, 145). Osteoblasts are mononucleated, specialized cells originating from MSCs and are primarily responsible for alveolar bone apposition (146). Osteocytes are derived from functional osteoblasts and are the dominant cells embedded in mineralized bone during the apposition process (146).

On the tension side, the periodontium (including the periodontal ligaments, alveolar bone, and cementum) undergoes bone deposition. Osteoblasts originating from MSCs form the osteoid or type I collagen matrix following mineralization (147). Meanwhile, alveolar bone resorption takes place on the compression side due to osteoclastic activity, resulting in irregular cavities in the bone, which are filled in by new bone from osteoblast activity (148). Orthodontic forces lead to increased blood vascular permeability and disarrangement of tissues in the periodontal ligament space. Subsequently, blood flow and periodontal tissue must adapt to the compression force. If they fail to adapt, tissue necrosis and hyalinization will occur (149). Force magnitude is considered to be related to the origination of osteoclastogenesis and the type of bone resorption. Applying light force leads to front resorption, the recruitment of hematopoietic progenitors from blood vessels in the periodontal ligament space, and cell and tissue preservation. Application of a heavy force causes hyalinization, cell death, tissue necrosis, and cell-free periodontal ligaments and adjacent alveolar bone zones, leading to delayed bone resorption. In human and animal studies, a heavy force causes more discomfort and pain than a light force (150–152).

### 6.2 The role of bone cells in nociceptor activation

During tooth movement, alveolar bone remodeling involves three major bone cells: osteoclasts, osteoblasts, and osteocytes. Compared to osteoblasts and osteocytes, osteoclasts have drawn more attention due to their association with nociceptors and pain, especially in skeletal diseases with increased bone resorption. In bone remodeling during tooth movement, osteoclasts may interact with nociceptive nerve terminals and contribute to pain-like behaviors differently. First, osteoclasts secrete large amounts of acid through vacuolar H^+^-ATPase, leading to bone matrix degradation. Nociceptors that innervate bone respond to acid through ASIC3 and TRPV1, and inhibition of these receptors attenuates experimental bone pain-related behaviors (153, 154). Second, osteoclasts produce pronociceptive factors, such as neurotrophic factors (155). Additionally, orthodontic pain is often related to periodontal ligament inflammation, where several pronociceptive mechanisms act synchronously (7). Unlike osteoclasts, the direct effect of osteoblasts and osteocytes on nociceptors and orthodontic pain is not well understood, even though they have been reported to participate in pathological bone pain, such as in osteoarthritis (156).

### 6.3 The role of nociceptors in regulating alveolar bone remodeling

Bone is richly innervated, and sensory and autonomic nerve fibers have been identified in both the periodontal ligament space and bone, with sensory fibers being associated with nociception and mechanoreception.

Recent studies have highlighted the regulatory role of the sensory nervous system in alveolar bone remodeling. While TRPV1-expressing nociceptive nerves contribute to pain during orthodontic forces, their roles in orthodontic tooth movement are not well-defined. More focus has been placed on studying the role of periodontal nociceptors in regulating alveolar bone remodeling in the context of periodontitis in mice. However, past studies investigating the control of bone remodeling and periodontal bone loss by nociceptive sensory nerves have produced contradictory results. The systemic injection of vanilloid compounds, such as capsaicin or RTX, either increases (157–159) or decreases (160–162) bone resorption. These discrepancies could be attributed to caveats in the neural manipulation adopted in past studies.

First, capsaicin and RTX are specific agonists of TRPV1. TRPV1 activation mediates Ca^2+^ influx, and an excessive Ca^2+^ influx often leads to the ablation of TRPV1 + nerve terminals and the soma (163, 164). The extent of activation and subsequent ablation by vanilloids depends on the dose, timing, location, frequency, route of administration, etc. Therefore, without thorough biological validations, it is difficult to conclude whether the effects of capsaicin are mediated by the activation or ablation of TRPV1 + afferent nerve terminals, leading to conflicting outcomes and complicating the interpretation of results. For example, the oral administration of capsaicin suppressed bone loss in a rodent periodontitis model, which was interpreted as having a protective role for TRPV1 + afferents (157). However, it is unclear whether this protective role was mediated by the activation or ablation of TRPV1 + nerve terminals. In humans, topical capsaicin on the gingiva increases neurogenic inflammation and the level of matrix metalloproteinase-8 (a periodontitis-associated protease) in crevicular fluid (165, 166), which is harmful rather than protective.

Second, intraperitoneal injection of RTX or capsaicin accelerates alveolar bone destruction in experimental periodontitis and reduces long bone density (157, 159). However, this treatment needs careful interpretation as it produces a deficiency of TRPV1 + neurons throughout the body and likely involves strong compensatory processes in the nervous system. Systemic treatment in neonatal animals is more problematic due to potential alterations in nervous system development (157, 158). Many studies have shown that systemic capsaicin injections in neonates increase sympathetic activity (167–171). Given the well-established role of sympathetic nerves in enhancing bone resorption (172–174), it is difficult to interpret the bone changes following systemic treatment of vanilloids as a pure effect of selective manipulation of sensory neurons. Indeed, systemic genetic ablation of tropomyosin receptor kinase A expressing neurons (largely overlapping with TRPV1 + neurons) increases serum norepinephrine and bone resorption, which can be reversed by propranolol (a β-adrenergic antagonist). This suggests the involvement of enhanced sympathetic activity (175). Likewise, nerve transection-induced aggravation of periodontitis (176) is difficult to interpret because transection of the inferior alveolar nerve produces neuropathic pain (177, 178), increasing sympathetic activity (179, 180).

Third, systemically injected vanilloids may directly affect bone cells, as TRPV1 is also known to be expressed in these cells (181–183). Conventional knockouts of TRPV1 have the same limitation, potentially affecting both neural and bone cells.

A recent study ablated TRPV1 + afferents by microinjecting RTX directly into the maxillary/ophthalmic region of a unilateral TG in adult mice to determine their role in periodontal bone remodeling (49). The resulting localized ablation of TRPV1 + afferents decreased bone loss in a mouse model of periodontitis (49), which is in contrast to the results obtained by the systemic injection of vanilloids (157, 158). This finding was further validated by localized chemogenetic silencing of TRPV1-lineage neurons using inhibitory designer receptors exclusively activated by designer drugs. Continuous functional silencing of TRPV1-expressing neurons after the induction of experimental periodontitis prevented the progression of bone loss in a mouse model of periodontitis (49). In these approaches, ablation or silencing was confined to the ipsilateral TG without altering TRPV1 afferents in the dorsal root ganglia or vagal ganglia, minimizing uncontrollable effects on nervous control. Circulating norepinephrine was not altered by the localized ablation of TRPV1-expressing TG neurons. Experimental periodontitis-induced increases in immune cell infiltration and pro-inflammatory cytokines were prevented in the mice using nociceptor ablation, which was accompanied by the reduced activation of osteoclasts in the alveolar bone. Interestingly, the ablation of TRPV1-expressing trigeminal nociceptors did not alter the periodontal microbiome within the ligature, suggesting TRPV1-expressing afferents enhance bone destruction in periodontitis by promoting hyperactive host responses in the periodontium (49). These findings support the theory that nociceptors magnify the host response and regulate bone loss in the periodontium without affecting host defenses. Although TRPV1-expressing afferents magnify alveolar bone remodeling in a periodontitis model, it is important to note that the contribution of nociceptive afferents to alveolar bone remodeling is context-dependent. Nociceptive nerves are protective in apical periodontitis following tooth pulp infection (184). Therefore, it is essential to determine the contribution of nociceptors on orthodontic tooth movement.

The activation of peptidergic nociceptors leads to Ca^2+^-dependent exocytosis of neuropeptides. SP and CGRP are the most abundant neuropeptides in sensory afferents, and both directly regulate bone cells *in vitro*. CGRP increases differentiation of osteoblasts and decreases osteoclastogenesis, thereby producing osteogenic effects (157, 185). In contrast, SP enhances osteogenesis and bone resorption *in vitro* (186–188). It increases the differentiation of osteoblasts and osteogenic activity at a physiologically relevant concentration (186). Consistently, SP knockouts exhibit reduced bone volume and trabecular number/thickness in the femur (189). At higher concentrations that are likely to be detected at the injury site, SP also enhances differentiation and the resorptive activity of osteoclasts *in vitro* (186, 190, 191). Thus, the consequences of SP signaling on pathological bone regulation *in vivo* is complicated to predict. Pharmacological or genetic inhibition of SP has shown both resorptive and osteogenic effects in osteoarthritis progression and fracture healing (189, 190, 192–194). Both CGRP and SP are detectable in human gingival crevicular fluid. The level of CGRP in crevicular fluid is lower at the site of periodontitis than at healthy sites (195–197). In contrast, the level of SP in the crevicular fluid is higher at sites of periodontitis than at healthy sites, and SP levels decrease after treatment (198, 199). However, due to the ambivalent role of SP, the consequences of increased SP for alveolar bone loss are uncertain. Interestingly, the deletion of tachykinin precursor 1, a gene that encodes SP, or the treatment of gingiva with an SP antagonist, significantly reduces bone loss in ligature-induced periodontitis. Whereas the deletion of calcitonin-related polypeptide alpha, a gene that encodes CGRP, has a marginal role in bone loss (72). Furthermore, exogenous SP, but not neurokinin A, induces a vigorous inflammatory response and osteoclast activation in alveolar bone and facilitates bone loss in ligature-induced periodontitis (72). SP knockouts show decreased immune cell infiltration and pro-inflammatory cytokines at the site of ligature, which recapitulates the findings from mice with nociceptor ablations (72). These findings suggest that SP plays significant roles in regulating host responses and bone resorption in ligature-induced periodontitis and that the resorptive effect of SP is sufficiently dominant to overcome the osteogenic activity of CGRP or SP.

Given the modest, or absence of, pain in periodontitis, the tooth movement model is better than the periodontitis model for determining the role of nociceptors in pain and the neural regulation of bone remodeling. Although the nociceptive regulation of orthodontic tooth movement has long been hypothesized (200), there is no strong evidence supporting the underlying mechanisms. The mechanistic contribution of neuropeptides in orthodontic tooth movement is not well known. Only one study has shown that the systemic administration of exogenous SP accelerates orthodontic tooth movement and promotes alveolar bone remodeling (136). The detailed regulatory mechanisms of orthodontic tooth movement by the nervous system, periodontal nociceptors, and nociceptive molecules should be determined in future studies.

### 6.4 The role of sympathetic nervous system in regulating alveolar bone remodeling

The critical role of the nervous system in regulating bone remodeling has been uncovered in recent decades (201). Besides sensory systems as discussed above, multiple other pathways, including adrenergic, dopaminergic, and serotonergic systems, are known to modulate bone remodeling. The roles of sympathetic nerves in enhancing bone resorption is well established (172–174). The autonomic fibers are mainly associated with supplying blood vessels in the periodontal ligament space and bone marrow and are concentrated in areas with high osteogenic activity (202). In the sympathetic nervous system, the β2-adrenergic receptor and dopamine receptors (203), expressed by osteoblasts and osteoclasts, are recognized as mediating the action of sympathetic nerves on bone remodeling. Sympathetic nerve terminals and local norepinephrine level in alveolar bone increase in periodontium after experimental tooth movement in mice (66, 204). Sympathectomy or β-adrenergic receptor antagonist propranolol decreased, whereas β-agonist isoproterenol increased experimental tooth movements in mice, suggesting that signaling from sympathetic nerve terminals within periodontium could regulate orthodontic tooth movements (66). These effects are mediated through Sympathetic signaling β2-adrenergic receptor expressed in osteoclasts and periodontal ligament cells (66, 205). Orthodontic tooth movement is also regulated through central modulation of the sympathetic nervous system, particularly through ventromedial hypothalamic nucleus, a region modulating sympathetic nervous system activity in periphery. Orthodontic tooth separations in patients increase the activation of hypothalamus (206). Experimental tooth movement in rodents increase tyrosine hydroxylase in ventromedial hypothalamic nucleus (204) and lesioning of the area suppresses tooth movement (207). Interestingly, the ablation of peripheral nociceptors by systemic injection of capsaicin reduces orthodontic tooth movements in mice, which is accompanied by decreased sympathetic nerve terminals in periodontium (207), suggesting potential interactions between nociceptive and sympathetic system for alveolar bone remodeling. Despite its roles in tooth movement, it is not known if sympathetic nervous system contributes to modulation of orthodontic pain.

On the other hand, the overall effect of parasympathetic activity on bone is likely to be anabolic, although various nicotinic or muscarinic acetylcholine receptors of the parasympathetic nerves have also been found in osteoclasts and osteoblasts (208). However, the role of parasympathetic activity on orthodontic tooth movement or pain is not known yet.

### 6.5 Implications of nociceptor-bone remodeling interactions in clinical orthodontics

Given the roles of nociceptors in alveolar bone remodeling, the activities of periodontal nociceptors and molecules enriched in periodontal nociceptors likely modulate orthodontic tooth movement. Therefore, the extent of tooth movement by orthodontic force can be interfered with when the activity of periodontal nociceptors is suppressed for treating pain. Although NSAIDs reduce orthodontic pain (8), they also reduce orthodontic tooth movement in humans and rats (209, 210). In contrast, acetaminophen does not inhibit tooth movement (211, 212), although the analgesic effects of acetaminophen and NSAIDs are comparable (8). Therefore, acetaminophen may be better for patients who suffer from orthodontic pain. To prevent tooth movement interference, the development of novel therapies for orthodontic pain should consider their effects on alveolar bone remodeling. For example, targeting TRPV1, ASIC3, or CGRP for orthodontic pain needs validation given that their inhibition does not interfere with tooth movement. Conversely, it would be ideal to develop methods to enhance tooth movement without inducing or increasing pain. Non-surgical interventions, such as light vibration, do not cause pain but fail to accelerate tooth movement (213, 214). The only method with evidence of accelerating tooth movement is corticotomy, which causes pain, discomfort, and functional impairments (215). Thus, better understanding and considering nociceptor-bone remodeling interactions is important for improving clinical orthodontics.

## 7 Conclusion

Despite decades of research, details on the mechanisms of orthodontic pain and tooth movement remain unclear. In particular, ongoing knowledge gaps and challenges remain substantial in terms of understanding the mechanistic interactions between the nervous and skeletal systems during tooth movement. A better understanding of the fundamental mechanisms of neuroskeletal interactions may improve orthodontic treatment by developing new methods to reduce pain and accelerate orthodontic tooth movement. Future studies in this area should meet clinical needs by developing new therapies for “big gains with no pain” in clinical orthodontics.
